# Quality of life, respiratory symptoms, and health care utilization 1 year following outpatient management of COVID-19: a prospective cohort study

**DOI:** 10.1038/s41598-022-17243-7

**Published:** 2022-07-29

**Authors:** Rachel K. Lim, Ryan Rosentreter, Yushi Chen, Rahul Mehta, Graham McLeod, Miranda Wan, Jonathan D. Krett, Yasamin Mahjoub, Angela Lee, Ilan Schwartz, Luanne Metz, Lawrence Richer, Eric Smith, Michael D. Hill, ALBERTA HOPE COVID-19 Collaborators, Rachel K. Lim, Luanne Metz, Michael D. Hill, Aravind Ganesh, Lawrence Richer, Ilan Schwartz, Aravind Ganesh

**Affiliations:** 1 Department of Medicine, Cumming School of Medicine, University of Calgary, Calgary, AB Canada; 2 Department of Clinical Neurosciences, Cumming School of Medicine, University of Calgary, HMRB 103, Heritage Medical Research Building, 3280 Hospital Dr. NW, Calgary, AB T2N 4Z6 Canada; 3 Department of Dentistry and Dental Hygiene, Faculty of Medicine and Dentistry, University of Alberta, Edmonton, AB Canada; 4 Department of Medicine, Faculty of Medicine and Dentistry, University of Alberta, Edmonton, AB Canada; 5 Department of Pediatrics, Faculty of Medicine and Dentistry, University of Alberta, Edmonton, AB Canada

**Keywords:** Microbiology, Signs and symptoms

## Abstract

The long-term impact of COVID-19 among those with mild infections is not well characterized. Among 81 adults who completed online assessments at 3- and 12-months following infection, quality of life scores did not significantly improve over time. Among 62 subjects who also completed telephone interviews, respiratory symptoms or exercise limitation were reported by 42% at a median follow-up of 387 days (IQR 251–402 days). Those with persistent respiratory symptoms scored lower on the EQ-5D visual analog score compared to those without. Persistent respiratory symptoms were associated with a lower likelihood of full-time employment at 1 year (aOR 0.09, 95%CI 0.01–0.91; P = 0.041). In an adjusted linear regression, persistent respiratory symptoms (P = 0.037) and female sex (P = 0.016) were both independent risks for increased visits to a primary care provider. This cohort study demonstrates that respiratory symptoms are frequent at 1 year following COVID-19 and more importantly, are associated with negative impacts on employment, quality of life, and health care utilization. Further research is needed to determine the pathophysiology and risk factors for persistent symptoms as well as optimal management strategies to improve the level of functioning and quality of life.

## Introduction

To date, severe acute respiratory syndrome coronavirus 2 (SARS-CoV-2) has caused more than 500 million infections globally^1^. Although many individuals with coronavirus-19 disease (COVID-19) have a complete recovery, a significant proportion of survivors experience long-term sequelae following the acute infection, loosely termed post COVID-19 condition or “long COVID”^2,3^. The natural course and burden of symptoms are poorly understood, particularly as a majority of studies focus on individuals who were hospitalized for COVID-19^2^. We prospectively assessed the 1-year outcomes among non-hospitalized survivors of COVID-19 using both a telephone and online questionnaire-based assessment.

Community-dwelling individuals with symptomatic, lab-confirmed SARS-CoV-2 infection in Alberta, Canada were recruited to a randomized controlled trial studying the efficacy of hydroxychloroquine (HCQ) in preventing severe disease between April and May 2020. In a province with a population of approximately 4.4 million, all non-hospitalized individuals with a positive molecular test were contacted for potential enrollment into the study. The methods of this trial are published elsewhere^4^. The trial was terminated early due to slow recruitment and the rise of potential safety concerns surrounding HCQ at the time; however, the trial cohort offered a unique opportunity to prospectively follow participants long term. Institutional ethics approval was obtained for this prospective cohort study and participants were contacted for informed consent and enrollment. The study was performed in accordance with relevant guidelines and regulations. We excluded four individuals who had been hospitalized during their acute illness. At 3 months and 12 months following their COVID-19 diagnosis, participants were asked to rate their quality of life using the EQ-5D-3L^5^ using an online link. Participants were phoned at 12 months post-COVID-19 for a structured interview about persistent symptoms or symptoms thought to be related to COVID-19, exercise ability, the number of visits to their family physician or emergency department since recovery, and employment status. Provincial healthcare records were reviewed to capture any hospitalizations or emergency department visits. Wilcoxon Rank Sum was used to compare continuous variables, Fisher’s exact test to compare proportions, and multivariable logistic regressions for adjusted comparisons of proportions. EQ-5D-3L ratings were converted to EQ-5D index scores using the Canadian value set^6^. All analyses were performed using STATA 16.1 (StataCorp LP, College Station, USA).

Among 84 participants who completed at least one assessment, the median time from COVID-19 diagnosis to final follow-up was 387 days (IQR 251 to 402 days). The mean age was 46.5 years (standard deviation 11.3) and 43% were male. Mean body mass index (BMI) was 29.5 kg/m^2^ (standard deviation 8.2). Cough was the most common reported symptom (n = 42, 79%) during acute COVID-19, followed by fever (n = 21, 40%), and dyspnea (n = 15, 28%). Hypertension (n = 13, 25%) and diabetes (n = 13, 25%) were the most common baseline comorbidities, while asthma was present in at least 17% of participants. Sixty-three subjects reported being either a former smoker or never smoker, while 11 were active smokers at baseline. Among 62 participants who completed the telephone symptom assessment at 1 year, 12 (19%) reported dyspnea and 13 (21%) reported cough at their 1-year follow-up. Using the modified Medical Research Council (mMRC) dyspnea scale^7^, a quarter of those with dyspnea reported an mMRC grade 2 or higher. If participants reported that their exercise tolerance had not returned to baseline yet, then this was considered as the presence of exercise intolerance. Persistent respiratory symptoms including reduced exercise tolerance were reported in 42% of the cohort at the final follow-up.

Participants reported a median of 2 visits (IQR 0 to 5) to their family physician for any reason since their initial COVID-19 diagnosis. Individuals with persistent respiratory symptoms visited their family physician more frequently compared to those without (median number of visits 3 versus 1, p = 0.0291). Although there was no control group in this study, it is worth noting that historical controls from a national survey reported that 54% of Canadians visited their family physician once a year or less in 2018^8^. Twelve individuals accounted for 15 visits to the emergency department (ED) since their diagnosis. There was no difference in ED visits between those with persistent respiratory symptoms and those without (p = 0.711).

As for daily function, 18 out of 62 (30%) stated that their exercise ability had not yet returned to their baseline prior to having COVID-19. In addition, patients reporting persistent respiratory symptoms were less likely to have full-time employment at 1-year follow-up compared to those without symptoms, even after accounting for pre-COVID-19 employment status (adjusted OR 0.09, 95%CI 0.01–0.91, p = 0.041). The EQ-5D-3L scores at 3 months and 12 months for 81 subjects are presented in Table 1. There was no statistically significant difference in scores between follow-ups. The median EQ-5D-3L scores were higher (indicating worse quality of life) among those with persistent respiratory symptoms compared to those without (p = 0.0278).

**Table 1:** Quality of life status (EQ-5D-3L) at 3 months and 12 months after COVID-19.

Domain of EQ-5D-3L	3 month follow-up N = 81	12 month follow-up N = 81	P-value
**Mobility**			
No problems	67 (83%)	69 (85%)	0.83
Some problems or unable to walk	14 (17%)	12 (15%)	
**Selfcare**			
No problems	80 (99%)	78 (96%)	0.62
Some problems or unable to wash or dress	1 (1%)	3 (4%)	
**Usual activities**			
No problems	67 (83%)	69 (85%)	0.83
Some problems or unable to perform	14 (17%)	12 (15%)	
**Pain**			
No pain or discomfort	48 (59%)	52 (64%)	0.63
Some or extreme pain or discomfort	33 (41%)	29 (36%)	
**Anxiety and depression**			
Not anxious or depressed	55 (68%)	55 (68%)	1.00
Moderately or extremely anxious or depressed	26 (32%)	26 (32%)	
Median EQ-5D-3L scores (IQR)	6 (5–7)	6 (5–7)	0.08
Mean EQ-5D Index Score^a^ (standard deviation)	0.859 (0.159)	0.881 (0.147)	0.06
EQ-VAS (0–100), mean (standard deviation)		76.3 (18.9)	

EQ-5D-3L, EuroQol Group Association five-domain, three-level questionnaire; EQ-VAS, EuroQol visual analogue scale.

^a^Health state index scores calculated from a Canadian value set^6^.

Adjusting for age and sex, a prior or current history of smoking was associated with 4.7-fold higher odds of having persistent respiratory symptoms compared to never-smokers (aOR 4.71, 95%CI 1.12–19.8, p = 0.035). A prior diagnosis of asthma or the presence of cough or dyspnea at the time of acute COVID-19 illness was not associated with persistent respiratory symptoms at 1 year. Patients with persistent respiratory symptoms were less likely to rate their quality of life satisfactorily (defined pragmatically as 75–100 on the EQ-5D visual analog scale, aOR 0.16, 95%CI 0.03–0.94, p = 0.043) after adjusting for age, sex, BMI, burden of initial symptoms, and prior treatment with HCQ. In a linear regression adjusted for age, BMI, prior HCQ treatment, and initial symptom burden, persistent respiratory symptoms (p = 0.037) and female sex (p = 0.016) were associated with increased visits to their family physician.

## Conclusions

There is growing evidence of considerable morbidity and reduced level of function among those with “mild” acute infections who did not require hospitalization^9,10^. This is concerning given that delayed recovery may lead to prolonged absence from work, school, or other activities, as well as a reduced quality of life. Our findings suggest that persistent respiratory symptoms including dyspnea, cough, and exercise intolerance were frequent at 1 year following infection by SARS-CoV-2 and that this was associated with lower employment levels, reduced quality of life and greater utilization of primary health care. Interestingly, the cohort’s median EQ-5D-3L scores did not differ between measurements. Although there was no significant difference between time points, this highlights the enduring impact of post COVID-19 condition, as many continued to report significant levels of anxiety, depression, pain, and mobility difficulties at 12 months.

Whereas our study has several strengths, including a prospective cohort design nested within a randomized controlled trial, with laboratory-confirmed COVID-19 for all participants, limitations include a relatively small sample, loss to follow-up, and a lack of a control group without COVID-19. Given that participants had to agree to be contacted for research and were already involved in a trial, there is a chance of recruitment bias. This study is one of a few to prospectively assess patients with milder outpatient illness at greater than a year following infection, providing useful information for health care providers managing “long COVID”. Further research is needed to determine the pathophysiology and risk factors for persistent symptoms as well as optimal management strategies to improve the level of functioning and quality of life.

### Ethics approval and consent to participate

Granted by the Conjoint Health Research Ethics Board at the University of Calgary (REB20-0790). Informed patient consent was obtained.

## Supplementary Information

Supplementary Information.

## Data Availability

The datasets analysed during the current study are not publicly available but are available from the corresponding author on reasonable request.
