# Supplementary material for: Quality of life, respiratory symptoms, and health care utilization 1 year following outpatient management of COVID-19: a prospective cohort study
Source: Sci Rep. 2022 Jul 29;12:12988. doi: 10.1038/s41598-022-17243-7 (PMC9334740; doi:10.1038/s41598-022-17243-7)
Supplement: Supplementary file 1 — Supplementary Information. [file 41598_2022_17243_MOESM1_ESM.docx]

Supplementary Information

**Members of the ALBERTA HOPE COVID-19 Collaborators**

**University of Alberta**: Nasreen Ahmad, Giiovanni Ferrara, Alim Hirji, Scott Jamieson, Wendy Johnston, Meghan Linsdell, Jennifer McCombe, Kirstie McDermot, Jeffrey Narayan, Manasi Rajagopal, Sarah Rathwell, Leka Ravindran, Lawrence Richer, Ilan Schwartz, Maeve Smith, Penny Smyth, Breanne Stewart, Taylor Strei, Diane Turner, Kelsey Tymkow, Sylvia van Os, Tanya Voth, Rick Watts, Maryna Yaskina; **University of Calgary**: Katayoun Alikhani, Jolene Allan, Mohammad Almekhlafi, Geri Anderson, Elizabeth Baguley, Megan Barber, Mari Boesen, Graziela Cerchiaro Farah, Anne Elaine Clarke, Carla Coffin, John Conly, Shelagh B. Coutts, Craig Doram, Katerina Downing, Tom Durnin, Brett Edwards, Aurora Fifi-Mah, Amanda Fisher, Aravind Ganesh, Virginia Gonzalez, Jamie Greenfield, Sheilah Heal, Alexis K. Hill, Michael D. Hill, Jia Hu, Kelly Johnston, Vikram Karnik, Sharanjit Kaur, Carol Kenney, Sundus Khan, Adam Kirton, Linda Knox, Katrina Koger, Alex Lemnaru, Rachel Lim, Ayla Lo, Natalie Lopez-Leacock, Jennifer McKeage, Paul MacMullan, Bijoy K. Menon, Luanne Metz, Christopher Mody, Kwadwo Mponponsuo, Jessica Ng, Blessing Odia, Milada Pajevic, Remo Pannacione, Michael Parkins, Lorina Platon, Sandra Rivest, Martha Rojas Zavala, Melanie Rosario, Karla J. Ryckborst, Shahana Safdar, Kayla Sage, Karla Sanchez, Supriya Save, Lisa Semeniuk, Ranjani Somayaji, Ericka Teleg, Alain Tremblay, Gloria Roldan Urgoiti, Jan Veenhuyzen, Adam Zedde; **Alberta Health Services**: Jeffrey Bakal, Shelly Bayley, Lauren Bemister, Lisa Bilston, Megan Brierley, Karen Brooks, Candice Cameron, Gillian Catena, Paola Charland, Quentin Collier, Ashley Drobot, Diana Horner, Reshma Karmali Alibhai, Daryl Lacombe, Marc Leduc, Delane Linkiewich, Debbie Mallet, Jatin Patel, Tristan Pidner, Pedro Reis, Mahnoush Rostami, Donna A. Smith, Michelle Stiphout, Nicole Tjepkema, Kelly van Camp, Rabia Wattoo, Becky Wong, Laurie Wright, Erik Youngson.
